# The Signal for Signaling, Found

**DOI:** 10.1371/journal.ppat.1000398

**Published:** 2009-04-24

**Authors:** Marcin Grynberg, Adam Godzik

**Affiliations:** 1 Institute of Biochemistry and Biophysics, Polish Academy of Sciences, Warsaw, Poland; 2 Burnham Institute for Medical Research, La Jolla, California, United States of America; The Rockefeller University, United States of America

An important task in the life of bacteria is to influence their environment, which for commensal, symbiotic, and pathogenic bacteria also includes their hosts. This is done primarily by secreting various sets of proteins (effectors), either simply to the outside environment or directly into host cells. This task is performed by specialized secretion systems that are complex, multi-protein molecular machines. While some of these systems are conserved across all domains of life, Gram-negative bacteria, with their two-layered membranes, face additional challenges and developed at least six different secretion systems. One of the best characterized systems is the type III secretion system (T3SS), discovered in 1994 by Rosqvist and colleagues in *Yersinia* [1]. It closely resembles and shares multiple elements with the flagellar apparatus, from which it probably evolved. We mostly associate this system with disease, such as plague, enterocolitis, whooping cough, pneumonia, and many other diseases that result from infections by Gram-negative species of *Yersinia*, *Salmonella*, *Shigella*, *Bordetella*, *Pseudomonas*, and other species, all of which use the T3SS. While secretion systems provide the means for invasion, the actual work is performed by the secreted proteins, or the effectors. To be successful, the right set of effectors must be expressed and bacteria must have a way to control which of the many proteins in the cytosol are being exported. In many secretion systems, this control is performed by specific signals on secreted proteins, usually the N-terminal signaling peptides that are recognized by the specialized components of the secretion system. Such a segregation may not only help in the secretion process, but may also ensure that only the actual effectors are secreted. In this context, the T3SS appeared to be somewhat of a puzzle, as T3SS effectors share no apparent common features. In many cases, close homologs of effector proteins of one species are not secreted in others. This has even led some to argue that the T3SS acts randomly, secreting proteins based on their abundance and proximity to the secretion apparatus. The question of identifying features of T3SS effectors is very important and goes beyond our fundamental interest in how such a system works. For many important pathogens we have not yet identified all T3SS effectors, making it possible that some important virulence factors, perhaps common to different diseases, are still unknown. For many less studied species we simply know nothing about possible effectors.

In this issue of *PLoS Pathogens*, two teams change this situation, presenting a novel methodology to recognize unknown effectors of the T3SS [2],[3]. Both groups used the advanced machine learning approach to identify a universal T3SS effector signature. The major breakthrough in both approaches was the use of machine learning algorithms able to recognize features that go beyond simple amino acid patterns and/or hydrophobicity profiles. Both approaches result in easy to use prediction algorithms and both groups predict scores of previously unknown (putative!) substrates of the T3SS; interestingly, they also predict substrates in species previously not studied for the presence of T3SS effectors, e.g., *Deinococci* or the pathogen *Chlamydia trachomatis*. In a fascinating sign of times, data analysis and verifiable predictions precede experimental work on many of the new systems, providing experimental groups in this field with both a challenge and a series of specific hypotheses and predictions.

Interestingly, many newly predicted effectors have no function assigned, suggesting that new classes of virulence factors may still remain to be characterized. Another intriguing question concerning the newly discovered effector signatures is where does such a generality come from? Arnold and colleagues [2] suggest that it is caused by convergent sequence adaptation of the signal peptide under the selective pressure rewarding the bacteria for optimizing their set of effectors. On the other hand, in a 2006 *PLoS Pathogens* article, Stavrinides and colleagues proposed a shuffling process of N-termini, coining a new term, “terminal reassortment” [4]. Arnold et al. advocate a syncretist model with both mechanisms acting at the same time. Last, but not least, recognition of common effector signatures suggests the existence of an as yet uncharacterized mechanism of effector recognition by the T3SS system. As with all good research, both papers, while answering some questions, raise many others and highlight new puzzles concerning the T3SS, as well as disease mechanisms of new and old pathogens.
